# Comparison of Invasive Interventions for Halting the Progression of Barrett’s Esophagus

**DOI:** 10.7759/cureus.108775

**Published:** 2026-05-13

**Authors:** Ranya Parekh, Aravind S Reddy, Umme Kalsoom, Rajesh C Varma, Arun Nair

**Affiliations:** 1 Medicine, Donald and Barbara Zucker School of Medicine at Hofstra/Northwell, Hempstead, USA; 2 Medicine, Gadag Institute of Medical Sciences, Mallasamudra, IND; 3 Medicine, Fatima Memorial College of Medicine and Dentistry, Lahore, PAK; 4 Pediatrics, Saint Peter's University Hospital, New Brunswick, USA; 5 Pediatric Hematology Oncology, Cohen Children's Medical Center, Queens, USA

**Keywords:** barrett’s dysplasia, barrett’s esophagus, cryotherapy ablation, endoscopic mucosal ablation, radiofrequency ablation (rfa)

## Abstract

Barrett’s esophagus (BE) is a premalignant condition that predisposes to esophageal adenocarcinoma, particularly in the presence of dysplasia. Once dysplastic change is identified, invasive interventions are often considered in efforts to prevent malignant progression. This review synthesizes evidence from an extended period of time to truly provide a comprehensive review regarding the efficacy, safety, and clinical utility of invasive therapies, including radiofrequency ablation (RFA), cryotherapy, argon plasma coagulation (APC), endoscopic mucosal resection (EMR), endoscopic submucosal dissection (ESD), photodynamic therapy, and esophagectomy. RFA remains the gold standard ablative therapy, achieving complete eradication of dysplasia and intestinal metaplasia with durable outcomes and a favorable safety profile. Cryotherapy provides comparable results and is especially valuable in patients with contraindications to thermal ablation or RFA-refractory disease. Of note, further head-to-head, larger clinical trials would be required to truly underline the more efficacious intervention. EMR and ESD allow removal of visible or nodular lesions and provide critical histopathologic staging, with ESD offering superior en bloc and curative resection rates but at greater procedural risk. APC achieves high eradication rates but with an elevated frequency of adverse events, limiting broad adoption. Current guidelines recommend endoscopic eradication therapy for high-grade dysplasia and emphasize individualized management for low-grade disease, underscoring the central role of RFA combined with resection when indicated. Remaining challenges include variability in operator expertise, uncertainty regarding long-term recurrence rates for newer therapies, and the need for standardized surveillance and biomarker-driven risk stratification. Collectively, the data support a patient-specific, stepwise strategy to halt disease progression while minimizing treatment-related morbidity.

## Introduction and background

Barrett’s esophagus (BE) is a premalignant condition characterized by the replacement of normal squamous epithelium in the distal esophagus with metaplastic, specialized intestinal-type columnar epithelium, a process often driven by chronic gastroesophageal reflux disease (GERD) [1]. This transformation is associated with an increased risk of progression to esophageal adenocarcinoma (EAC), a malignancy with poor prognosis and rising incidence in Western populations [2-4]. Although only a subset of patients with BE progress to high-grade dysplasia (HGD) or EAC, the development of dysplasia represents a critical clinical threshold at which surveillance and therapeutic intervention become essential.

The prevalence of BE is estimated at 1-2% in the general population, increasing to 5-15% among individuals with chronic GERD [5]. It is more common in Western populations, with a marked male predominance and higher prevalence among Caucasian individuals [1,4]. Established risk factors include long-standing reflux, central obesity, smoking, and advancing age [2,6]. While proton pump inhibitors (PPIs) effectively control reflux symptoms and may reduce mucosal injury, they do not eliminate the risk of malignant progression, underscoring the need for effective surveillance and intervention strategies [1].

Initial management of BE centers on acid suppression with PPIs to control reflux and reduce mucosal injury [1]. The absence of dysplasia (low-grade dysplasia, LGD), HGD, or intramucosal carcinoma necessitates intervention [7]. Current guidelines recommend endoscopic eradication therapy (EET) for dysplastic BE, favoring interventional approaches over surveillance alone [8,9].

Invasive management strategies include endoscopic ablative therapies, such as radiofrequency ablation (RFA), cryotherapy, and photodynamic therapy (PDT), and endoscopic resection techniques, including endoscopic mucosal resection (EMR) and endoscopic submucosal dissection (ESD). Resection techniques enable the removal of visible lesions, provide histopathologic staging, and are frequently combined with ablative modalities in a stepwise approach [6,7]. Esophagectomy remains a definitive yet highly invasive option, reserved for select patients with advanced or refractory disease [9].

Despite advances in endoscopic therapy, uncertainty persists regarding optimal treatment selection, sequencing, and long-term durability. This review aims to evaluate the efficacy, safety, and clinical outcomes of invasive interventions in BE, with a focus on preventing progression to EAC and identifying areas for future research.

## Review

Methods

The study design of this paper is that of a literature review. The databases used to collect articles included PubMed and Google Scholar, with a filter applied to retrieve free full-text English-language articles from 2007 to 2025. A wider year range was applied to gain a broader sense of the development of interventions for BE, and all study designs were included in the search. Search terms included a combination of “Barrett’s esophagus”, “dysplasia”, “radiofrequency ablation”, “cryotherapy”, “argon plasma coagulation”, “endoscopic mucosal resection”, “endoscopic submucosal dissection”, “photodynamic therapy”, and “esophagectomy”. Studies were eligible for inclusion if they were published in English, involved human subjects, and/or reported clinical outcomes related to efficacy, safety, or recurrence of invasive therapies for BE. All study designs were considered, including randomized controlled trials, cohort studies, and meta-analyses. The pooled estimates should be interpreted with caution, given the potential statistical heterogeneity between included studies. Articles not directly relevant to invasive management of BE or lacking sufficient clinical data were excluded. Identified studies were screened for relevance by three independent reviewers, and discrepancies and study selection were resolved through discussion and consensus. Data from selected studies were extracted and organized using Microsoft Excel (Microsoft Corporation, Redmond, WA, USA). As this was a narrative review, formal risk-of-bias assessment and quantitative synthesis were not performed. The final manuscript was developed through a comprehensive review among all authors to ensure consistency with the study objectives.

Global variations in the incidence of BE

Although BE is increasingly recognized worldwide, its incidence and prevalence display striking geographic, ethnic, and temporal variability, reflecting the impact of genetic predisposition, environmental exposures, and health system practices. In Western nations, particularly North America and Northern Europe, population-based endoscopic studies estimate a BE prevalence of 5-15% among individuals undergoing endoscopy for reflux symptoms, with overall population prevalence around 1-2% [10,11]. By contrast, the prevalence in East Asia, including Japan, Korea, and China, has historically been reported at <2% even among patients with chronic GERD, despite high rates of reflux disease in those regions [12]. This paradox has been partly attributed to the higher prevalence of *Helicobacter pylori* infection in Asia, which is inversely associated with BE, possibly due to hypochlorhydria and reduced acid exposure [13]. However, as *H. pylori *prevalence declines with urbanization, recent studies suggest a rising trend of BE in younger Asian cohorts, particularly in urban centers, mirroring patterns previously observed in the West [12,14].

Ethnic and racial differences within countries further underscore the heterogeneity of BE. In the United States, BE and EAC are disproportionately more common in White populations compared to African American, Hispanic, and Asian populations [15]. These disparities may reflect genetic polymorphisms in inflammatory or metabolic pathways, differences in obesity and central adiposity, dietary risk factors, or disparities in access to endoscopy [16]. Male sex and advancing age remain the most consistent demographic risk factors globally, with a male-to-female ratio of almost 3:1 and peak incidence in the sixth decade of life [17].

Temporal trends highlight additional complexity. Over the past three decades, the incidence of BE and EAC has increased steadily in Western populations, particularly among younger men, even as peptic ulcer disease has declined [18]. In contrast, regions such as South America, the Middle East, and South Asia remain understudied, with sparse population-based data limiting reliable estimates of incidence [19]. This paucity of data hampers the development of globally harmonized surveillance and treatment strategies.

From the standpoint of invasive interventions, these variations have significant implications. Because most EET data are derived from Western cohorts, global differences in BE prevalence and EAC epidemiology may limit the generalizability of trial findings and cost-effectiveness assumptions. Conversely, in regions where BE remains rare and EAC is less tightly linked to reflux (e.g., East Asia, where squamous cell carcinoma predominates), widespread invasive intervention may not be cost-effective. Understanding these global epidemiologic differences is therefore critical for tailoring surveillance and treatment paradigms and for informing the generalizability of clinical trial data that predominantly originate from Western cohorts.

Clinical indications for invasive intervention

The therapeutic escalation from surveillance to invasive management in BE is predicated on the degree of dysplasia, the presence or absence of visible mucosal abnormalities, and the confirmation of histopathological diagnosis by expert gastrointestinal pathologists. For patients with nondysplastic BE, invasive intervention is not indicated, as the risk of neoplastic progression remains low, with pooled annual incidence rates of EAC reported at 0.2-0.5% [20,21]. Consequently, guideline committees uniformly recommend optimization of acid suppression and structured endoscopic surveillance in this cohort rather than prophylactic ablation [7].

The presence of dysplasia, however, represents a clinical threshold for intervention. In LGD, considerable interobserver variability complicates histological interpretation, with diagnostic concordance among expert pathologists remaining poor in several prospective evaluations [22,23]. Nevertheless, when LGD is confirmed in duplicate endoscopies and verified by more than one expert pathologist, progression rates to HGD or adenocarcinoma rise substantially, with estimates ranging from 9% to 13% at five years [23]. Randomized controlled trials have demonstrated that ablation in this context significantly reduces the incidence of neoplastic progression compared with surveillance alone, justifying its inclusion in current guideline recommendations [24].

Meta-analyses suggest that LGD progresses to HGD or adenocarcinoma at an annual rate of 0.5-1%, while HGD carries a risk exceeding 6% per year [25]. Patients with HGD or intramucosal carcinoma constitute the most unequivocal indication for EET. Endoscopic resection is mandated in the presence of visible or nodular mucosal abnormalities, both to achieve complete local excision and to provide accurate histopathological staging. Large multicenter cohorts have shown that EMR alters the histological diagnosis in nearly one-third of cases, underscoring the inadequacy of biopsy sampling alone for staging early neoplasia [24]. Furthermore, resection of visible abnormalities prior to ablation mitigates the risk of residual sub-squamous neoplasia, given the superficial effect of thermal or cryogenic ablation modalities [26]. Following successful resection, ablation of the remaining Barrett’s segment markedly decreases the risk of recurrent dysplasia or intestinal metaplasia, with pooled recurrence rates as low as 4-8% in long-term series [27-29].

Flat HGD, in the absence of visible lesions, is less common but equally significant. Flat HGD without visible lesions should be diagnosed cautiously, as subtle visible neoplasia may be missed without careful high-definition inspection by experienced endoscopists. Accordingly, expert consensus requires histological confirmation on at least two consecutive endoscopies prior to definitive ablation [7]. Intramucosal carcinoma, if confined to the mucosa, remains amenable to curative endoscopic resection followed by ablation of residual Barrett’s mucosa. However, evidence of submucosal invasion typically precludes endoscopic therapy and necessitates surgical resection, as the risk of lymphatic involvement exceeds the therapeutic reach of endoscopic modalities [30].

Invasive intervention in BE is justified in three principal contexts: confirmed LGD after expert review, HGD with or without visible lesions, and intramucosal carcinoma confined to the mucosa. Outside these categories, particularly in nondysplastic BE or indefinite dysplasia, ablation is not currently supported, and structured surveillance remains the standard of care. This stratified approach reflects the delicate balance between mitigating the risk of malignant progression and avoiding overtreatment in a population where absolute cancer risk remains relatively low.

Types of invasive interventions

The prospective invasive management for dysplastic BE comprises several modalities, each distinguished by its mechanism, efficacy in achieving complete eradication of dysplasia (CE-D) or intestinal metaplasia (CE-IM), accompanying complications, and their respective strengths and limitations. A critical appraisal of these options informs best practices in EET.

RFA employs controlled thermal energy delivered via balloon or focal electrode to eradicate metaplastic epithelium [31]. A meta-analysis of nineteen studies indicates that RFA achieves pooled CE-D and CE-IM rates of approximately 91% (95% CI: 87-95) and 78% (95% CI: 70-86), respectively, with recurrences reported in about 13% of cases (95% CI) [9-18,32,33]. Longitudinal data corroborate these results: one cohort reported 89.8% CE-IM and 90.4% CE-D, durable over five years with stringent follow-up; adverse events included strictures (5.4%), bleeding (0.8%), and no perforations; five-year survival was 91.9% [34]. Recent studies highlight that optimized energy dosimetry and circumferential balloon catheters have reduced recurrence and stricture formation [35]. RFA’s advantages include high efficacy, durable outcomes, and a favorable safety profile. Treatment failure or incomplete response may occur, particularly in patients with long-segment BE, uncontrolled reflux, large hiatal hernias, or persistent intestinal metaplasia [36-38]. Overall, RFA remains the gold standard for first-line ablative therapy.

Cryotherapy, via spray or cryoballoon, induces apoptosis through extreme cold. A systematic review and meta-analysis encompassing 23 studies (n = 1,604) demonstrated comparable CE-D and CE-IM rates with those of RFA, pooled at 84.2% (95% CI: 79.1-89.3) and 64.1% (95% CI: 49.2-79.0), respectively, with recurrence in 8.3% and adverse events in 14.5% (strictures ~6.5%) [39,40]. A meta-analysis directly comparing cryotherapy and RFA confirmed similar efficacy and recurrence profiles [40]. However, long-term durability data are limited compared with RFA, with recurrence rates reported as high as 25% at three years [41]. A prospective cryoballoon series achieved technical success in 94.7% of lesions, underscoring its practical feasibility [42,43]. In a small cohort, cryotherapy achieved 100% eradication, with minor pain reported in 4% and 9% recurrence [44]. Cryotherapy’s strengths lie in its comparable efficacy and utility when thermal injury or stricture risk is a concern, while limitations include heterogeneity in outcomes and less robust long-term data.

Hybrid argon plasma coagulation (H-APC) and standard argon plasma coagulation (APC) represent noncontact thermal ablation using ionized argon gas. A network meta-analysis including RFA, APC, and PDT situated APC as achieving the highest rate of CE-IM (86.8%), whereas PDT showed the highest CE-D (94.1%) [45]. However, adverse events for APC were higher (~22.5%), although strictures were relatively low, around 1.7% [45]. One comparative long-term study reported BE eradication in 82.9% of H-APC versus 74.2% in RFA after extended follow-up [46]. While APC offers strong efficacy for IM eradication, the notably elevated complication rate and limited widespread use temper its appeal [47].

EMR and ESD are resection-based techniques, particularly advantageous for visible or nodular lesions. A large meta-analysis contrasting EMR and ESD for early Barrett’s neoplasia found that ESD afforded significantly higher en bloc resection (OR ~25.96) and R0 resection (OR ~5.10), along with lower recurrence in non-RFA patients (OR ~0.22), albeit with increased adverse events including bleeding, strictures, and perforation [48-51]. Another systematic review found ESD’s en bloc and R0 resection rates superior, with curative resection tending higher and recurrence lower compared to EMR [52]. Observational studies reported en bloc (80-100%), complete resection (up to 96%) with adverse event rates such as perforation (<1.5%), bleeding (~1.7%), and strictures (~11.6%) [53]. These approaches provide histopathologic precision and efficacy, particularly when integrated with ablation. However, adverse events such as bleeding (5-10%) and stricture formation (up to 20% after ESD) remain significant considerations in clinical decision-making [54] and require considerable endoscopic expertise, as well as carrying elevated procedural risk.

PDT employs photosensitizing agents activated by light to destroy dysplastic tissue. Although historically used and associated with high CE-D rates (up to ~94%), PDT’s use has declined significantly due to adverse events, including photosensitivity reactions and stricture formation; direct comparative efficacy has largely been surpassed by RFA and cryotherapy [55]. Currently, PDT is reserved for rare cases where ablation and resection are contraindicated or unavailable.

Esophagectomy, the surgical removal of the esophagus, remains the most definitive treatment, particularly when invasion extends beyond the mucosa or when endoscopic modalities fail. However, it is highly invasive, associated with significant perioperative morbidity and mortality, lengthy recovery periods, and profound impact on quality of life. Consequently, it is reserved for specific contexts where endoscopic therapies are inappropriate or unsuccessful [56].

Table 1 summarizes the comparison between the aforementioned interventions.

**Table 1:** Summary and comparison of invasive interventions in BE AE, adverse events; APC, argon plasma coagulation; BE, Barrett’s esophagus; CE-D, complete eradication of dysplasia; CE-IM, complete eradication of intestinal metaplasia; EET, endoscopic eradication therapy; EMR, endoscopic mucosal resection; ESD, endoscopic submucosal dissection; H-APC, hybrid argon plasma coagulation; HGD, high-grade dysplasia; LGD, low-grade dysplasia; PDT, photodynamic therapy; QoL, quality of life; RFA, radiofrequency ablation Data adapted from references [31-56]

Therapy	Mechanism	Efficacy (CE-D /CE-IM)	Recurrence	Complications	Clinical role/notes
RFA	Thermal ablation with balloon/focal electrode	CE-D ≈ 90%; CE-IM ≈ 80-90%	~10-15% at five years	Strictures (5%), bleeding (<1%), no perforations	Gold standard; first-line for LGD/HGD; durable outcomes
Cryotherapy	Extreme cold (spray or balloon) → necrosis	CE-D ≈ 84%; CE-IM ≈ 64% (meta-analysis)	8-10%	Strictures ~6.5%, pain (mild)	Useful for RFA failure, stricture-prone esophagus, or contraindication to thermal therapy
APC/H-APC	Ionized argon plasma → superficial ablation	CE-IM ≈ 86%; CE-D lower than RFA	Variable	Adverse events up to 20%, strictures rare (~1-2%)	Promising efficacy but limited by safety; less widely adopted
EMR	Resection of visible/nodular lesion	Lower en bloc rates vs. ESD; effective for focal disease	Recurrence higher vs. ESD	Bleeding, perforation (<2%), stricture	First-line for visible lesions; often followed by RFA
ESD	En bloc resection of the lesion + submucosa	En bloc up to 100%; curative resection higher than EMR	Recurrence lower vs. EMR	Higher AE: strictures (10-12%), bleeding, perforation (<1-2%)	Best for larger/nodular lesions; requires expertise, mostly tertiary centers
PDT	Photosensitizer + light → tissue necrosis	CE-D up to 94%	Moderate	Strictures, photosensitivity	Rarely used; supplanted by RFA/cryotherapy
Esophagectomy	Surgical removal of the esophagus	Near-curative if localized	N/A	High morbidity/mortality, QoL decline	Reserved for invasive disease or failed EET

In summary, RFA continues to anchor endoscopic treatment for dysplastic BE due to its high eradication rates, durability, and safety [57]. Cryotherapy emerges as a robust alternative when RFA is contraindicated or unsuccessful. APC/H-APC offer promising IM eradication but are hampered by elevated adverse events. EMR/ESD are indispensable for resection and histologic evaluation of focal lesions, though more complex and risky. PDT and esophagectomy now serve niche roles, invoked in specialized or advanced cases.

Scientific basis of therapeutic modalities in BE

RFA

The biological basis of EET in BE lies in the selective destruction of metaplastic mucosa followed by the regeneration of neosquamous epithelium. Different modalities achieve this through distinct energy-tissue interactions that exploit the columnar epithelium's relative susceptibility to injury.

RFA delivers controlled thermal energy (60-100°C), inducing coagulative necrosis of dysplastic glands within the lamina propria while preserving deeper structures. Post-ablation healing promotes repopulation by squamous progenitor cells from adjacent epithelium and submucosal glands. Experimental data suggest RFA induces proptosis via protein denaturation and caspase activation, with reductions in dysplastic clones and inflammatory signaling, supporting durable re-epithelization [58,59].

APC and Cryotherapy

H-APC produces superficial coagulative injury (~0.5-1 mm depth), making it well suited for residual metaplasia. Cryotherapy induces tissue injury through freeze-thaw cycles, resulting in intracellular ice formation, vascular stasis, and apoptosis. Preservation of extracellular matrix architecture may facilitate healing with less fibrosis [60]. Experimental models also suggest upregulation of stress-response and angiogenic pathways that support mucosal repair [61].

Endoscopic Resection Techniques

Endoscopic resection techniques, including EMR and ESD, address focal neoplasia by removing dysplastic tissue and enabling histologic staging to exclude submucosal invasion. ESD allows en bloc resection with improved margin assessment, reducing residual neoplastic tissue that may persist after surface ablation [62]. Healing occurs through squamous re-epithelialization from adjacent mucosa.

PDT

PDT induces apoptosis by generating reactive oxygen species upon photoactivation of a photosensitizer. However, nonselective tissue injury contributes to higher rates of strictures and photosensitivity, limiting its current use [63].

Molecular Determinants of Treatment Response

Treatment response appears to be influenced by underlying molecular features. Dysplastic mucosa with TP53 mutations, aneuploidy, or chromosomal instability may demonstrate increased resistance to therapy and higher recurrence rates [64]. In contrast, less genomically altered tissue may achieve more durable eradication, highlighting the potential role of molecular profiling in guiding future therapeutic strategies.

Long-term outcomes, recurrence, and emerging therapies

While invasive interventions for BE have revolutionized disease management, their long-term durability and evolving adjunctive strategies warrant careful appraisal. EET, incorporating ER for nodular disease followed by RFA for flat mucosa, remains the standard of care. Longitudinal studies demonstrate that RFA achieves CE-IM in 78-90% of patients, with sustained eradication of dysplasia in up to 93% [35,65]. However, recurrence of BE after initial eradication remains a significant clinical challenge, with reported rates of recurrent intestinal metaplasia ranging from 10% to 20% at five years and recurrent dysplasia or carcinoma in 3-5% [66,67].

Risk factors for recurrence include long-segment BE, baseline presence of HGD, incomplete initial ablation, and male sex [68]. Recurrence often arises at the gastroesophageal junction, a region that is technically challenging to eradicate due to its anatomical complexity and ongoing exposure to acid or bile. These data reinforce the principle that eradication therapy should not be viewed as curative but rather as disease-modifying, necessitating structured endoscopic surveillance even in patients with apparent complete remission. The optimal surveillance interval remains debated, though recent modeling studies suggest that risk-stratified surveillance based on baseline histology and segment length may improve cost-effectiveness without compromising cancer prevention [69].

Alternative ablative modalities, including cryoablation, have demonstrated comparable short-term efficacy but less robust durability data. Prospective multicenter cohorts suggest that cryotherapy achieves CE-IM in 75-85% of patients, though recurrence rates approach 25% within three years [70]. It is important to note, however, that most cryotherapy data is found in RFA cohorts, which can indicate a degree of selection bias. Its role is often as salvage therapy in patients with RFA failure, highlighting the need for comparative trials with longer follow-up. EMR or ESD, while effective for visible lesions, carries higher risks of stricturing and does not address the entire at-risk mucosal field, predisposing to metachronous neoplasia unless combined with ablation [54]. Surgical esophagectomy, though now rarely employed for noninvasive disease, remains the definitive salvage option in cases of invasive carcinoma or refractory recurrence, albeit with substantial morbidity and mortality [71].

Incomplete or aberrant healing can reestablish a permissive niche for metaplasia. If residual Barrett’s glands survive within the submucosa, they can reemerge under persistent reflux stress, driven by inflammatory cytokines and aberrant Notch signaling [72]. Moreover, chronic PPI-resistant bile exposure impairs squamous differentiation and perpetuates columnar regeneration. This underscores the importance of acid suppression and anti-inflammatory therapy to maintain neosquamous integrity.

Tissue engineering approaches and biomaterial scaffolds are being explored to modulate post-ablation healing. Bioactive hydrogels releasing EGF or anti-fibrotic agents have shown promise in preclinical models for enhancing epithelialization and reducing strictures [73]. The identification of resident esophageal progenitors and their niche regulators opens possibilities for pharmacologic manipulation, for example, targeting Wnt or YAP/TAZ pathways to promote organized squamous regeneration while suppressing metaplastic reprogramming.

Collectively, translational insights into esophageal regeneration underscore the biologic continuum between injury, repair, and neoplastic risk. By modulating these pathways, next-generation endoscopic therapies may not only eradicate dysplasia but also actively steer mucosal healing toward a stable, non-metaplastic state.

Novel approaches aim to enhance durability and personalize therapy. Second-generation cryoablation platforms with balloon- or spray-based systems allow for more uniform ablation and appear to induce less postprocedural pain, with early reports suggesting improved mucosal healing and fewer recurrences [74]. Hybrid techniques combining resection with subsequent cryoablation or APC are being evaluated to optimize field eradication [75].

AI represents a particularly transformative frontier. Computer-aided detection and diagnosis algorithms now demonstrate high sensitivity for identifying subtle dysplasia in BE, reducing interobserver variability and improving lesion targeting [76]. Early-phase studies suggest AI integration may reduce sampling error, decrease procedure times, and facilitate real-time guidance for ablation completeness. While clinical adoption is in its infancy, these tools may soon redefine procedural standards.

The integration of molecular biomarkers into clinical practice promises to shift BE management from a uniform to a precision-based paradigm. Tissue-based assays such as TissueCypher may improve risk stratification beyond histology alone, but routine incorporation into clinical practice remains limited by evolving evidence and guideline uncertainty [77]. Additional biomarkers, including p53 immunohistochemistry, DNA methylation panels, and transcriptomic classifiers, have shown strong associations with neoplastic progression [64,78]. Importantly, biomarker-driven models may enable tailoring of surveillance intensity and more judicious use of invasive therapies, reducing overtreatment while identifying high-risk patients earlier.

The convergence of endoscopic innovation, AI-assisted imaging, and molecular biomarker stratification suggests a future in which invasive interventions are not applied uniformly but rather integrated into precision algorithms. In this framework, invasive eradication would be reserved for those with a demonstrated biologic risk of progression, while low-risk individuals could be safely monitored with less intensive surveillance. Such an approach promises to maximize the cancer-preventive benefit of invasive therapies while minimizing recurrence, cost, and morbidity.

Discussion

The management of dysplastic BE has undergone a notable paradigm shift, with EET now firmly established as the standard of care in patients with HGD and intramucosal carcinoma. The 2024 American Gastroenterological Association (AGA) guideline strongly recommends EET for BE with HGD and conditionally suggests EET for LGD, while surveillance remains appropriate for selected LGD patients after shared decision-making [79].

From an evaluative perspective, RFA demonstrates the most favorable balance between benefit and risk and remains the cornerstone of endoscopic therapy for dysplastic BE. Eradication rates consistently exceed 90% for dysplasia and approach 80-90% for intestinal metaplasia, with recurrence rates generally under 15% [80,81]. These outcomes have been validated across large registries, randomized trials, and long-term follow-up studies, providing a level of evidence not yet matched by other modalities [82,83]. However, its efficacy is not uniform across all patient subgroups. Incomplete response or treatment failure has been observed in patients with long-segment disease, complex mucosal architecture, or adverse reflux-related factors [84]. Rather than undermining its role, these limitations emphasize the need for a tailored, multimodal approach, incorporating resection for visible lesions and alternative ablative strategies in selected cases.

Cryotherapy, although achieving comparable aggregate eradication rates, illustrates the complexity of interpreting therapeutic equivalence. Meta-analyses report pooled complete CE-D rates near 84% and CE-IM rates around 64%, which appear close to RFA [40,85]. Yet heterogeneity across studies, shorter follow-up, and variations in technique make direct comparisons problematic. In practice, cryotherapy is often used in patients who have failed RFA, where its value may be underestimated in aggregated statistics. Thus, its role is not as a universal first-line therapy but as a complementary tool in a multimodal strategy. The implication is that guideline hierarchies of therapy may oversimplify what is, in reality, a nuanced and context-dependent treatment landscape.

Endoscopic resection (EMR/ESD) further underscores this principle. Their primary value lies not in replacing ablation but in enabling accurate histopathologic staging and the treatment of focal neoplasia [80]. Meta-analyses clearly show that ESD offers superior en bloc resection and lower recurrence compared with EMR [52]. However, the higher risk of perforation and stricture, along with the requirement for specialized expertise, limits its applicability outside tertiary centers [53,55]. This divergence between efficacy under controlled conditions and feasibility in real-world practice illustrates a recurrent theme in BE management: optimal strategies are as dependent on local expertise and infrastructure as on published trial results [56,57]. In this sense, cost-benefit analyses of ESD versus EMR are not limited to clinical outcomes but extend to training, procedure time, and institutional resources.

Alternative modalities such as H-APC or conventional APC also reveal this tension between efficacy and practicality. Although CE-IM rates above 85% have been reported, adverse event rates approaching 20% substantially reduce clinical utility [85]. For these therapies, the issue is less one of biological plausibility and more one of acceptability in practice, given that safer and equally effective options exist. The same rationale explains the declining role of PDT, in which stricture formation and photosensitivity outweigh the oncologic benefit [55]. These examples demonstrate that clinical adoption is not merely dictated by efficacy endpoints but also by the overall therapeutic index.

Surgical esophagectomy represents the other extreme. Its near-curative potential comes at the cost of significant perioperative morbidity, mortality, and impaired quality of life [70]. Consequently, it is now reserved for advanced disease or failed endoscopic therapy. The critical takeaway here is not simply that surgery has been supplanted, but that its risks highlight the success of endoscopic modalities in shifting the treatment paradigm toward organ preservation. Taken together, these comparisons suggest that the optimal management of BE is not defined by a single therapy but by a stepwise, stratified approach [86]. RFA serves as the backbone, but its limitations necessitate adjunctive resection and, in refractory cases, alternative ablative modalities such as cryotherapy. APC and PDT illustrate how promising therapies may falter due to safety concerns, while esophagectomy remains indispensable for select advanced cases. This layered approach aligns with the principle of tailoring intervention to lesion characteristics, patient comorbidities, and institutional capacity [87,88].

Beyond modality-specific considerations, several broader themes emerge. First, recurrence rates, even after apparently complete eradication-underscore the importance of surveillance. Estimates suggest a 10-15% recurrence rate over five years, regardless of modality [89-92]. This persistent risk highlights the premalignant biology of BE and the need for durable follow-up strategies. Second, the success of these interventions is closely tied to operator expertise, especially for technically demanding procedures such as ESD. Guidelines appropriately recommend centralizing complex care in high-volume centers [92-94], but this raises equity concerns in access to advanced therapy. Finally, future directions increasingly point toward biomarker-driven stratification. Recent guideline updates acknowledge the potential of tissue-based assays, such as the TissueCypher test, to identify patients at higher risk of progression [95,96]. Integrating such tools into clinical practice may refine surveillance and intervention thresholds, moving management toward a more individualized model.

The therapeutic approaches for dysplastic BE reflect a careful consideration of efficacy, safety, durability, and feasibility. RFA retains primacy, but its imperfections invite the use of complementary modalities. Cryotherapy and EMR/ESD extend the armamentarium by addressing specific clinical scenarios rather than competing directly with RFA [97]. APC and PDT, while historically or experimentally relevant, remain limited by safety concerns, while esophagectomy serves as a definitive salvage approach. Cost-effectiveness analyses consistently support RFA over surveillance alone for dysplastic BE, particularly when progression risk exceeds 0.5% per year. Post-eradication recurrence highlights the need for standardized surveillance protocols, yet optimal intervals remain debated. Furthermore, most trials have been conducted in expert centers, raising concerns about generalizability to community practice. Future research should focus on refining surveillance strategies, integrating tissue-based biomarkers to identify high-risk patients, and optimizing the sequencing of ablation and resection modalities. The overarching trend is clear: management has shifted from invasive surgery to minimally invasive endoscopic therapy, but optimal outcomes require tailoring interventions to patient and lesion characteristics, delivered within guideline frameworks and supported by long-term surveillance.

## Conclusions

Management of dysplastic BE has shifted decisively toward minimally invasive, endoscopic approaches. RFA remains the gold standard therapy, supported by high eradication rates, durability, and a favorable safety profile. Cryotherapy offers similar efficacy and is particularly valuable in patients who are unsuitable for thermal ablation. EMR and ESD provide indispensable diagnostic and therapeutic capabilities for nodular lesions, often in combination with ablative therapy. APC and related modalities show promise but are limited by complication rates, while PDT and esophagectomy are reserved for specialized or refractory cases. Current AGA guidelines reinforce this hierarchy, recommending EET for HGD and selective use for LGD, alongside lesion-specific therapy and multidisciplinary expertise. The available data support a tailored, stepwise approach that maximizes eradication of dysplasia while minimizing patient morbidity. Ongoing advances in molecular diagnostics, device technology, and AI-assisted endoscopy are expected to further refine risk stratification and expand minimally invasive options for BE management. Nonetheless, gaps remain in long-term outcomes, surveillance strategies, and the integration of predictive biomarkers. Future work should refine patient selection, optimize procedural sequencing, and explore personalized follow-up strategies. Ultimately, effective management of BE depends on aligning high-quality evidence with guideline-based care, expert execution, and patient-centered decision-making.
